# Six-month survival of critically ill patients with HIV-related disease and tuberculosis: a retrospective study

**DOI:** 10.1186/s12879-016-1644-6

**Published:** 2016-06-10

**Authors:** Ana Carla Pecego, Rodrigo T. Amancio, Camila Ribeiro, Emersom C. Mesquita, Denise M. Medeiros, José Cerbino, Beatriz Grinsztejn, Fernando A. Bozza, Andre M. Japiassu

**Affiliations:** Intensive Care Clinical Research Laboratory, National Institute of Infectious Diseases (NIID), Av Brasil 4365, Manguinhos, Rio de Janeiro, RJ 21045-900 Brazil; STD/AIDS Clinical Research Laboratory, National Institute of Infectious Diseases (NIID), Av Brasil 4365, Manguinhos, Rio de Janeiro, RJ 21045-900 Brazil; Instituto D’Or de Pesquisa e Ensino, Rua Diniz Cordeiro, n° 30, Botafogo, Rio de Janeiro, RJ 22281-100 Brazil

**Keywords:** HIV, AIDS, Tuberculosis, Critical care, Patient outcome

## Abstract

**Background:**

Tuberculosis is one of the leading causes of death from infectious diseases worldwide, mainly after the human immunodeficiency virus (HIV) epidemics. Patient with HIV-related illness are more likely to present with severe TB due to immunosuppression. Very few studies have explored HIV/TB co-infection in critically ill patients. The goal of this study was to analyze factors associated with long-term mortality in critically ill patient with HIV-related disease coinfected with TB.

**Methods:**

We conducted a retrospective study in an infectious disease reference center in Brazil that included all patient with HIV-related illness admitted to the ICU with laboratory-confirmed tuberculosis from March 2007 until June 2012. Clinical and laboratory variables were analyzed based on six-month survival.

**Results:**

Forty-four patients with HIV-related illness with a confirmed diagnosis of tuberculosis were analyzed. The six-month mortality was 52 % (23 patients). The main causes of admission were respiratory failure (41 %), severe sepsis/septic shock (32 %) and coma/torpor (14 %). The median time between HIV diagnosis and ICU admission was 5 (1–60) months, and 41 % of patients received their HIV infection diagnosis ≤ 30 days before admission. The median CD4 count was 72 (IQR: 23–136) cells/mm^3^. The clinical presentation was pulmonary tuberculosis in 22 patients (50 %) and disseminated TB in 20 patients (45.5 %). No aspect of TB diagnosis or treatment was different between survivors and nonsurvivors. Neurological dysfunction was more prevalent among nonsurvivors (43 % vs. 14 %, *p* = 0.04). The nadir CD4 cell count lower than 50 cells/mm^3^ was independently associated with Six-month mortality (hazard ratio 4.58 [1.64–12.74], *p* < 0.01), while HIV diagnosis less than three months after positive serology was protective (hazard ratio 0.27, CI 95 % [0.10–0.72], *p* = 0.01).

**Conclusion:**

The Six-month mortality of HIV critically ill patients with TB coinfection is high and strongly associated with the nadir CD4 cell count less than 50 cels/mm^3^.

## Background

Tuberculosis remains a global health issue and the leading causes of death from infectious diseases worldwide, mainly after the human immunodeficiency virus (HIV) epidemics. The main population affected by TB mortality is HIV-positive individuals. Throughout 2014, 1.5 million deaths occurred, of which 0.4 million were among seropositive patients [1]. Tuberculosis prophylaxis and treatment is still suboptimal in patients with HIV coinfection in developing countries, even with novel diagnostic tests and antimicrobial agents [1].

Fifteen percent of new TB cases in Brazil are among HIV-infected individuals, which reinforces the recommendation for systematic TB screening for hospitalized patients with HIV-related disease [2–4]. HIV-positive persons that present with advanced immunosuppression more frequently present with hematogenous dissemination of the bacillus and multiorgan involvement, and are frequently smear-negative, requiring blood cultures and invasive procedures for diagnosis [5–8].

HIV-TB coinfection accounts for 26.6 % of TB mortality rate [1], and the subgroup of patients that require ICU has an even worse prognosis [4]. Respiratory failure is the most frequent cause of ICU admission of patients with HIV-related illness, and TB is associated with distinct prevalence according to the geographic location where the studies are performed [9–11].

Very few studies have explored HIV/TB coinfection in critically ill patients [9, 12]. Disseminated presentation of TB, low serum albumin levels, delayed diagnosis and multilobar lung involvement have been identified as markers of poor prognosis [8, 9, 13, 14]. Additionally, there is uncertainty about absorption of anti-tuberculosis medications, and drug interactions with antiretroviral therapy are still of concern, mainly when rifampicin and protease inhibitors are prescribed simultaneously [8, 10].

The purpose of this study was to identify factors associated with six-month mortality of critically ill patients with HIV-related disease, with confirmed diagnosis of TB in a major referral center for HIV care in Rio de Janeiro, Brazil.

## Methods

### Design and setting

This is a retrospective study conducted at the ICU of the National Institute of Infectious Disease at Oswaldo Cruz Foundation (FIOCRUZ) in Rio de Janeiro, Brazil. Our institution has provided care to HIV-positive patients since 1986. There are currently over 3.300 HIV-positive adult under active follow-up at Outpatient Clinic. Cohort characteristics have been published elsewhere [15, 16]. We admit approximately 60–70 patients with HIV-related disease per year in the ICU.

### Definitions, selection of participants and data collection

#### Study procedures

All files of patients with HIV-related disease admitted to the ICU who received TB treatment from March 2007 to June 2012 were retrospectively reviewed. Only patients with laboratory confirmed tuberculosis were included in this report. The follow-up for six-month survival was checked through consulting electronic data chart of ambulatory and hospital assistance.

Clinical variables related to HIV infection were: cART exposure, time from HIV diagnosis until ICU admission, nadir CD4 cell count, CD4 cell count and HIV viral load recorded at ICU admission or collected in the previous three months prior to hospital admission. Variables for tuberculosis infection were: clinical presentation of tuberculosis (pulmonary or disseminated), clinical specimens that allowed microbiological TB confirmation, anti-tuberculosis drugs prescribed while staying in the ICU, and *Mycobacterium tuberculosis* resistance diagnosed by culture.

The severity of illness was evaluated using the Simplified Acute Physiological Score (SAPS) II and Sequential Organ Failure Assessment score (SOFA). The SAPS II score assigns points based on age, type of admission (scheduled surgical, unscheduled surgical, or medical), 12 physiological variables, and three underlying disease variable (AIDS, metastatic cancer, and hematologic malignancy); this score expresses the level of acute severity along with age and the presence of these severe comorbidities in the first 24 h after ICU admission [18]. The score varies from 0 to 163 points, and the higher the SAPS II value, the less is the probability of survival. SOFA score is a semi-quantitative score of six organ dysfunctions, named cardiovascular, respiratory, neurological, hematological, hepatic and renal dysfunctions [19]. It classifies a range of six organ dysfunctions: cardiovascular (hypotension or use of vasopressors); respiratory (PaO2/FiO2 rate); neurological (Glasgow Coma Scale); renal (serum creatinine level and/or daily diuresis); hepatic (serum bilirubin level); and hematological (platelets level). The score goes from 0 to 24 points, and it can be calculated on a daily basis, in order to evaluate the patient’s improvement or worsening. SOFA also gives qualitative information about the Multiple Organ Dysfunction Syndrome (MODS). For ease of interpretation, we also evaluated the initial SOFA score (day 1), since one score higher than 11 points is associated with less than 20 % chance of survival [20]. We also evaluated the use of vasoactive drugs, non-invasive and invasive ventilation support, and hemodialysis. We classified all ICU admission diagnoses as: respiratory insufficiency, severe sepsis or septic shock, coma/torpor and miscellaneous [21].

Demographic and clinical data were categorized according to the survival after six months. The results were displayed as frequency (percentage), median values and interquartile range. If a patient had multiple ICU admissions, only the first was included in this analysis. The ICU team, infectious disease specialist and family members in accordance with local practices shared decisions regarding withholding or withdrawal of treatment.

#### Case definition

Tuberculosis was considered confirmed if the bacteriologic case definition was met: direct visualization of acid-fast bacilli (Ziehl-Neelsen stain), (“smear positive”), or a positive culture for *Mycobacterium tuberculosis* in Lowenstein-Jensen or liquid medium (MIGT). A histopathological result consistent with *Tuberculosis* in a patient with high clinical suspicion was also considered a confirmed case. Patients were excluded if central nervous system (CNS) involvement was highly suggested by CT scan and/or CNS fluid exams, since CNS tuberculosis infection carries a much higher mortality per se and could overscore on CGS [22]. Besides the high mortality rate it could distort the analysis of the group, and therefore it should be analyzed separately.

Combined antiretroviral treatment (cART) exposure before ICU entry meant any cART use regardless of adherence to the treatment. We defined cART as administration of three antiretroviral drugs belonging to at least two classes (i.e., nucleoside reverse-transcriptase inhibitor, non-nucleoside reverse-transcriptase inhibitor, protease inhibitor, integrase inhibitor).

In our population, we had two scenarios of the administration of TB drugs while in the ICU, according to the hemodynamic status. In the case of hemodynamic unstable patients, TB drugs were delivered intravenously; fluoroquinolones and aminoglycosides were the drugs of choice. When the patient was hemodynamically stable, TB drugs could be delivered per enteral or oral route; the combination of Rifampicin (R), Isoniazid (H), Pyrazinamide (P), Ethambutol (E) were the preferred drugs given by oral/enteral route.

Disseminated TB was defined when: thoracic X-ray or CT scan showed a pattern that indicated dissemination; two non-contiguous sites were involved in accordance with WHO 2013; or *Mycobacterium tuberculosis* was recovered from the bloodstream [1].

Drug resistance was defined as resistant to ≥ 1 drug [17], and Multidrug-resistant tuberculosis (MDR) defined as TB caused by strains of Mycobacterium tuberculosis that are resistant to at least isoniazid and rifampicin [23].

#### Laboratory procedures

Resistance towards anti-TB drugs was determined by culture methods using liquid media or Lowenstein-Jensen slants.

#### Statistical analysis

We analyzed the clinical and laboratory variables according to six-month survival after ICU admission using GraphPad Prism version 6.0 for MAC OS X (GraphPad Software, San Diego, CA, USA) and Epi Info™ version 7.1 (Centers for Disease Control, Atlanta, GA, USA). The numerical demographic variables were expressed as the median and the interquartile range (IQ 25 –75 %). All of the numerical variables were tested for normality using the Kolmogorov-Smirnov test. We compared continuous variables using a *t*-test (parametric distribution) and the Mann–Whitney *U* test (nonparametric distribution). The categorical variables were compared using the chi-squared test and Fisher’s exact test. We categorized two numerical variables, since the dispersion was wide: CD4 cell count (below 50 cells/mm3) and time since HIV diagnosis (recent HIV means less than three months after positive HIV serology). We performed the survival analysis with Cox proportional hazards to analyze factors associated with six-month survival, selecting variables with p values lower than 0.2 in the univariate comparison. A Kaplan-Meier curve was constructed based on independent factors associated with six-month survival.

## Results

### Clinical characteristics of patient with HIV-related illness with tuberculosis in the ICU

**Fig. 1:**
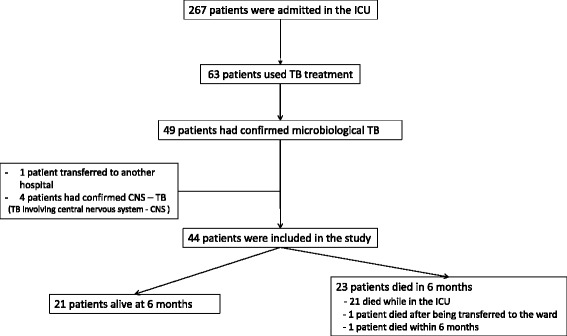
Flow diagram for study inclusion

During the five years study period, 267 HIV/AIDS patients were admitted to the ICU and 63 received tuberculostatic treatment. Among these, 49 had laboratory-confirmed tuberculosis; four patients were excluded because of confirmed TB CNS involvement and one patient was transferred to another hospital. A total of 44 patients with HIV-related disease with confirmed TB were included in the study. All patients were followed up to six months after ICU discharge. The ICU mortality was 48 % (21 patients), one patient died after being transferred to the hospital ward and one patient died within the following six months after ICU discharge (Fig. 1).

The median age was 34 (IQR: 19–44.5) years, with a predominance of males (70 %) (Table 1). The main reasons for ICU admission were respiratory failure (41 %), severe sepsis/septic shock (32 %), coma/torpor (14 %), and miscellaneous (13 %). The SAPS II score was 47 (IQR: 37–55) points, and the SOFA score on day 1 of the ICU stay was 4 (IQR: 1–8) points. Mechanical ventilation was needed in 63 % of patients.

**Table 1:** Characteristics critically ill HIV patients coinfected with TB (laboratory-confirmed) diagnosis, including the characteristics of Tuberculosis (TB) presentation and treatment and the prevalence of organ dysfunctions at ICU admission (*N* = 44)

	HIV/AIDS and Tuberculosis (*N* = 44)	Survivors (*N* = 21)	Non-survivors (*N* = 23)	*p* value
Age (years)	34 (19.25–44.5)	32 (27–42.5)	36 (30–46)	0.15
Gender (male)	31 (70 %)	14 (67 %)	17 (74 %)	0.74
Nadir CD4^a^	54 (17–96)	73 (30–177)	23 (14–84)	0.04
Viral load (×10^3^ copies/mm^3)b^	113,637 (2,995–365,663)	35,149 (212.3–300,184)	169,133 (35,405–419,382)	0.13
Length of time since HIV diagnosis (months)	5 (1–60)	1 (1–16)	28 (1–72)	0.03
cART use while in the ICU	17 (38.6 %)	10 (48 %)	7 (30 %)	0.35
Mechanical ventilation use on ICU day 1	21 (48 %)	8 (42 %)	13 (52 %)	0.56
Vasoactive drug use on ICU day 1	12 (26 %)	5 (24 %)	7 (27 %)	0.99
SAPS II score (points)	47 (37–55)	48 (37–57)	46 (38–54)	0.27
SOFA score on day 1 (points)	4 (1–8)	1 (0–7)	5 (2–8)	0.08
Isolated TB pulmonary presentation	22 (50 %)	10 (48 %)	12 (52 %)	1.00
Disseminated TB presentation	22 (50 %)	11 (52 %)	11 (48 %)	1.00
TB treatment before ICU admission	25 (57 %)	11 (52 %)	14 (61 %)	0.76
TB treatment				
- only by enteral drugs	16 (36 %)	10 (48 %)	6 (26 %)	0.21
- adding intravenous drugs	28 (64 %)	11 (52 %)	17 (74 %)	
TB treatment: Δ ICU admission	0 (-5–+21)	-5 (-23–+2)	-5 (-21–0)	0.09
TB treatment before ICU	25 (57 %)	11 (52.4 %)	14 (61 %)	0.76
Organ Dysfunctions				
Cardiovascular	18 (41 %)	7 (33.3 %)	11 (48 %)	0.37
Respiratory	15 (34 %)	5 (24 %)	10 (43 %)	0.21
Renal	14 (32 %)	5 (24 %)	9 (39 %)	0.34
Neurological	13 (29 %)	3 (14 %)	10 (43 %)	0.04
Hepatic	9 (20 %)	5 (24 %)	4 (17 %)	0.71
Hematological	9 (20 %)	3 (14 %)	6 (26 %)	0.46

Comparison between survivors and nonsurvivors for six-month survival. Results were expressed as the median and the interquartile interval

^a^data available for 39 patients

^b^data available for 35 patients

Δ:time from starting TB treatment and beeing admitted to ICU. Negative (-) mean prior to ICU admission and positive (+) means after ICU admission

The main differences between survivors and nonsurvivors for six-month mortality were: nadir CD4 cell count, median time length between HIV-infection diagnoses and ICU admission, SOFA score, and neurological dysfunction. Twenty-one (45 %) patients had a recent HIV diagnosis (within three months before hospital admission). The most recent median CD4 cell count was 36 [IQR: 15–80] in nonsurvivors vs. 100 [IQR: 58–243] cells/mm^3^ in survivors (*p* = 0.01); with a median time between CD4 cell count and ICU admission – 0.5 months (IQR: -2.25–0).

Twenty-five (57 %) patients were previously exposed to cART, and only 4 (9 %) presented with undetectable viral load (less than 50 copies/ml). Combined ART was used in 17 (39 %) patients while in the ICU, with no statistical difference in both, hospital mortality (52 % vs 29 %, *p* = 0.23) and in six months survival (48 % vs 30 % *p* = 0.35). There was no statiscally difference in six-month mortality between survivors and nonsurvivors using cART while in the ICU (48 % vs 30 %, *p* = 0.35). There was no difference in six-month mortality between survivors and nonsurvivors using cART during the first week of ICU stay (38 % vs 17 %, *p* = 0.09), or during the 2nd week (33 % vs 35 %, *p* = 0.78). None of the patients presented IRIS nor revealed an unmasked infection while taking cART in the ICU.

### Tuberculosis diagnosis and treatment

No aspect of TB diagnosis or treatment was different in survivors or nonsurvivors. Twenty-two pulmonary TB cases (50 %) and 22 disseminated (50 %) TB cases were equally distributed between the 2 groups (Table 1). Tuberculosis was diagnosed from respiratory samples in the majority of cases. Thirty-two (73 %) patients were diagnosed through direct visualization of the bacillus using the Ziehl-Neelsen technique (27 in the sputum and 5 in the bronchoalveolar lavage); the diagnosis was only made through culture of respiratory specimens in 5 (11 %) patients. Bacteremia due to *Mycobacterium tuberculosis* was found in 6 (14 %) patients. Eleven (25 %) patients required invasive procedures for TB diagnosis, such as organ biopsy. The main TB regimens prescribed were per oral or enteral routes in 16 patients, while the addition of intravenous drugs (mostly aminoglycosides and quinolones) was chosen in 28 patients. The combination of oral/enteral drugs was composed by HRZ (*n* = 8) or HRZE (*n* = 8) schemes. Intravenous fluoroquinolones and aminoglycosides were prescribed in 27 (61 %) and 22 (50 %) patients, respectively.

We identified resistance to *M. tuberculosis* in specimen cultures from 5 (11 %) patients: resistance to streptomycin (*n* = 4), isoniazid (*n* = 2), and rifampin (*n* = 1). From these five patients, only one patient with isolated resistance to streptomycin survived; the other four patients with a pattern of resistance died. None of 44 patients included had multidrug resistant Mycobacterium tuberculosis strains.

#### Organ dysfunctions

Although not statistically significant on day 1 of ICU admission, the SOFA score was higher among non-survivors (*p* = 0.08) (Table 1). For this reason, we decided to determine if any of the six organ dysfunctions could be associated with six-month survival. Only neurological dysfunction was statistically different: 10 (43 %) nonsurvivors vs. 3 (14 %) survivors presented with neurological dysfunction, *p* = 0.04. Neurological dysfunction was described as GCS of 13–14 points (somnolence) in 9 patients, and GCS of 8–10 points (torpor) in four patients. One patient had seizures, two patients had gait disturbances and one patient had paresthesia. None had neck stiffness, delirium [24] or intracranial hypertension. Of the four patients with a GCS of less than 12 points, all had head CT scans and three had liquor studies: three patients were diagnosed with neurotoxoplasmosis and one patient had normal head CT scan.

#### Six-month outcome

**Fig. 2:**
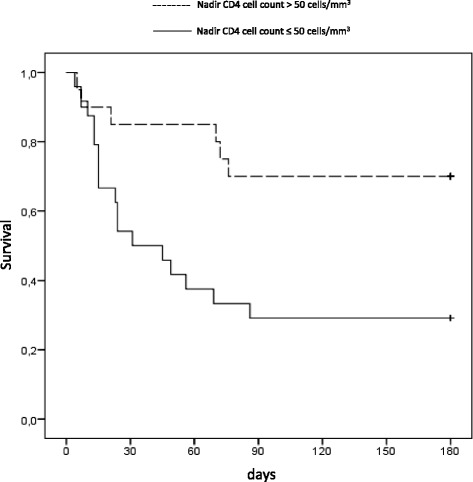
Kaplan-Meier plot of six-month survival in critically ill patients with HIV-related disease and tuberculosis, based on nadir CD4 cell count less than 50 cells/mm^3^. Log-rank test *p* = 0.006

The nadir CD4 cell counts lower than 50 cells/mm^3^ was independently associated with six-month mortality (hazard ratio 4.58 [1.64–12.74], *p* = 0.004), while recent HIV diagnosis (less than three months after positive serology) was protective (hazard ratio 0.27, CI 95 % [0.10–0.72], *p* = 0.01) (Table 2). We observed a lower six-month survival in patients with nadir CD4 cell counts lower than 50 cells/mm^3^ (log rank test *p* = 0.006) (Fig. 2).

**Table 2:** Cox proportional analysis and adjusted hazard ratio for six-month mortality (95 % CI) of critically ill patients with HIV-related disease with tuberculosis (*N* = 44)

Variable	Adjusted Hazard ratio for six-month mortality	*p* value
Nadir CD4 cell count less than 50 cells/mm^3^	4.58 [1.64–12.74]	0.004
Age at inclusion	1.03 [0.99–1.08]	0.07
SOFA score at ICU admisson	1.09 [0.97–1.22]	0.13
Recent HIV diagnosis at admission^a^	0.27 [0.10–0.72]	0.009

^a^recent HIV diagnosis: less than three months of HIV positive serology

## Discussion

The coinfection HIV-TB presented a high long-term mortality in our cohort. The nadir CD4 cell count was the independent marker for six-month mortality, while a HIV diagnosis up to three months before hospital admission was protective among these severely ill HIV-TB patients. No aspect of tuberculosis presentation or treatment influenced the outcome. Among the organ dysfunctions, neurological dysfunction was more frequent in nonsurvivors, even after the exclusion of those patients with primary TB CNS involvement.

### HIV infection and critical illness

Since the beginning of the HIV epidemic, respiratory failure has been the main reason for ICU admission with the spectrum of etiological agents changing according to the geographic region and cART access [25–28]. Influenza, pneumococcal vaccination and TB background prevalence also influence the main causes of respiratory disease in this population [29–32]. Tuberculosis is highly prevalent in Brazil, mainly in Rio de Janeiro, and the HIV infection epidemic has made TB a common cause of opportunistic infections responsible for AIDS diagnosis in our population [2, 33]. Rio de Janeiro has one of the worst scenarios in regards to the Tuberculosis epidemic in Brazil. In 2012, 10,649 new tuberculosis cases were diagnosed in Rio de Janeiro State, with an incidence rate of 65.6/100.000 person-years, a rate much higher than the overall country incidence rate of 35.8/100.000 person-years. The mortality rate among TB patients from Rio de Janeiro is the second highest in the entire country, reaching 5.1/100.000 person-years.

Respiratory failure is the main reason for ICU admission among pulmonary HIV-TB patients [8–10], as we described in our population, even though 45 % of patients present with disseminated tuberculosis. Isolated extrapulmonary disease was uncommon (5 %).

Severe immunosuppression caused by HIV infection is a known risk factor for tuberculosis [7]. As immunosuppression evolves, the TB disease spectrum changes from its classic pulmonary presentation of unilateral upper lobe infiltrate to a broad range of nonspecific signs and symptoms related to hematogenic spread of the bacillus. The myriad of presentations of TB in the population with HIV-related disease is associated with challenges in establishing the diagnosis and higher mortality [33]. Our high proportion of disseminated TB (45 %) is due to the advanced immunodeficiency status of our population, with median CD4 cell counts of 72 cells/mm^3^, which were lower than previously reported (109 and 112 CD4 cells/mm^3^) [28,29]. CD4 cell count is thought to be an unreliable predictor of ICU mortality, according to Morris and colleagues, when compared to other known risk factors, such as mechanical ventilation, low serum albumin and APACHE II score [11, 34–37]. However, others authors believe that CD4 cell count does have a correlation with a poorer prognosis [25, 38–40]. Low CD4 counts (<200 cells/mm^3^) have been associated with ICU mortality in HIV patients with TB [41]. In the present study, we found that patients with higher nadir CD4 cell counts were more likely to be alive six months after hospital discharge. These data are supported by the idea that CD4 cell counts at ICU admission are a better marker of long-term prognosis [42]. We preferred nadir than recent CD4 cell count because opportunistic infections and concomitant acute infections can lower the CD4 cell count [45]. Alongside with the low nadir CD4 cell count, we wanted to emphasize that we also observed a low median CD4 count close to ICU admission meaning that our population represents a profoundly immunocompromised population. Also, nadir CD4 cell count it is better marker of prognosis and disease evolution in HIV infected population [43, 44]. We also found that recent HIV diagnosis was associated with protective effect for long term survival. This variable should be evaluated with caution: there is not an accurate correlation of the moment of first HIV positive serology and the time since HIV infection began. Some patients are admitted with recent HIV diagnosis and very low nadir CD4 count, although they were previously less exposed to bacterial and multilple oportunistic infections and to the HIV infection per se, than their peers with long known HIV diagnosis and non-adherence to cART. This state of chronic immunosuppression by HIV and opportunistic infections may have contributed to a lower survival rate in this population, as shown previously in a heterogeneous group of studies with and without cART influence [11, 30, 58].

As show earlier from our institution’s cohort, the HIV diagnosis frequently occurs during an advanced immunodeficiency state, which can be associated with a high prevalence of tuberculosis [46]. Earlier diagnosis is fundamental to diminish the complications of the advanced immunodeficiency.

Delivering cART in critically ill patients is still controversial. The limited availability of intravenous medications, potential drug-to-drug interactions, erratic gastrointestinal absorption and the risk of unmasking infections and IRIS are considerations in the setting of critically ill patient with HIV-related disease [37, 38, 53]. The administration of cART did not influence short- or long-term survival in our study, but we cannot conclude that cART administration in the ICU is not beneficial since it was not possible to assure adherence of all patients taking cART after hospital discharge, which is fundamental to the success of the treatment. In the present study, we did not find Immune Reconstitution Inflammatory Syndrome (IRIS) criteria in any case, nor did we find any severe adverse reactions attributed to cART.

### Characteristics of tuberculosis infection

The recovery of *M. tuberculosis* from blood cultures is suggestive of disseminated TB, and we found bacteremia in 14 % of patients. This is a common etiologic agent of sepsis in population with HIV-related disease, as observed in two published works by our group and other groups [11, 29, 43–49]. Research suggests that patients with disseminated and extra-pulmonary forms of tuberculosis may represent a special group that may be associated with worse outcomes, including greater risk for the tuberculosis immune reconstitution inflammatory syndrome [50]. TB treatment in the acute phase of critically ill patients can often be suboptimal [51, 52] because first line drugs are only available in oral formulations. One could suppose that intravenous treatment could improve outcomes (fluoroquinolones and aminoglycosides). However, in this case series, we found a high proportion of critically ill patients using rifampin, isoniazid and pyrazinamide, and half of our patients received intravenous quinolones and/or aminoglycosides as the first treatment for TB with no difference in six-month survival.

### Risk factors for long-term mortality

The six-month mortality observed in our study was high (52 %), but lower than reported from other retrospective studies involving patients with TB admitted to the ICU (hospital mortality 22–67 %) [10, 41, 54]. Another interesting point was that the disseminated form of TB had a similar mortality to pulmonary presentation (45 % and 50 %, respectively). This may be different from another study, which enrolled 46 HIV-TB patients, including suspected cases of TB, although we included only confirmed cases [10]. Finally, improvements in the care of critically ill patients, such as implementation of protective ventilator strategies with low tidal volume, conservative fluid strategy for Acute Respiratory Distress Syndrome (ARDS), and early sepsis goals, have added benefits and have recently had an impact on lower mortality rates [28, 34, 55].

This study highlights neurological dysfunction as being an important marker for mortality among critically ill HIV-TB patients. These findings cannot be attributed to encephalitis or meningitis that is primarily caused by TB. We excluded CNS involvement (encephalitis or meningitis), which carries a poorer prognosis [56, 57]. All patients with GCS ≤ 11 had a lumbar puncture performed. The majority of our neurological findings were mild; most of them were classified as torpor. As shown previously, low GCS at admission can be a reliable predictor of ICU mortality [35, 58]. In our analysis, neither SOFA nor SAPS II was able to predict survival. Another two studies have found correlation between higher SAPS II and ICU mortality [35, 51]. Zahar et al. investigated laboratory-confirmed tuberculosis cases, 38 % were patients with HIV-related, all with respiratory failure, and the majority with a pulmonary presentation; higher SAPS II (approximately 45 points) and the presence of more than one organ failure were significantly associated with mortality [51]. Meybeck et al. showed that SAPS II was significantly higher in nonsurvivors (approximately 55 points) with an odds ratio of 1.05 per point [35]. The SAPS II score was similar between six-month survivors and nonsurvivors in our study, while SOFA scores on the day of ICU admission trended towards being higher in nonsurvivors (*p* = 0.08).

### Limitations of the study

We realize that our study has several limitations. First, it is a retrospective single-center study conducted in a specialized infectious disease hospital, and these findings cannot be extrapolated to other centers. The fact that we did not find IRIS in our population may due to the retrospective nature of the study since we did not have clear definitions since the beginning of our cohort. The limited number of patients (44 patients) is another limitation of our study since we are exploring a very specific population. However, the results here presented should increase awareness to important factors that may contribute to a better management of HIV-TB patients in the ICU, and for pointing to future researches. Regarding the neurological findings, due to its retrospective nature, we were not able to detect broader neurological involvement, such as delirium and intracranial hypertension [58]. Delirium screening using the Confusion Assessment Method (CAM-ICU) assessment is not a routine practice at our institution [24].

## Conclusions

The six-month mortality of HIV critically ill patients with TB coinfection is high and strongly associated with the nadir CD4 cell count less than 50 cels/mm^3^. Neurological dysfunction may be associated with poor survival, even without primary CNS involvement.

## Abbreviations

APACHE, acute physiological assessment and chronic health evaluation; ARDS, acute respiratory distress syndrome; CAM, confusion assessment method; cART, combined antirretroviral therapy; CGS, coma glasgow scale; CNS, central nervous system; CT, computed tomography; HIV, human immunideficiency virus; ICU, intesive care unit; IRIS, immune reconstitution inflammatory syndrome; MDR, multidrug-resistant; MODS, multiple organ dysfunction syndrome; SAPS, simplified acute physiological score; SOFA, sequenctial organ failure assessment score; TB, tuberculosis
